# Multifrequency Induced-Charge Electroosmosis

**DOI:** 10.3390/mi10070447

**Published:** 2019-07-03

**Authors:** Kai Du, Jingni Song, Weiyu Liu, Ye Tao, Yukun Ren

**Affiliations:** 1 School of Electronics and Control Engineering, and School of Highway, Chang’an University, Middle-Section of Nan’er Huan Road, Xi’an 710064, China; 2 State Key Laboratory of Robotics and System, Harbin Institute of Technology, West Da-Zhi Street 92, Harbin 150001, China

**Keywords:** multifrequency induced-charge electroosmosis, simultaneous pumping and convective mixing, dual-Fourier-mode AC forcing, traveling-wave/standing-wave AC electroosmosis, microfluidics

## Abstract

We present herein a unique concept of multifrequency induced-charge electroosmosis (MICEO) actuated directly on driving electrode arrays, for highly-efficient simultaneous transport and convective mixing of fluidic samples in microscale ducts. MICEO delicately combines transversal AC electroosmotic vortex flow, and axial traveling-wave electroosmotic pump motion under external dual-Fourier-mode AC electric fields. The synthetic flow field associated with MICEO is mathematically analyzed under thin layer limit, and the particle tracing experiment with a special powering technique validates the effectiveness of this physical phenomenon. Meanwhile, the simulation results with a full-scale 3D computation model demonstrate its robust dual-functionality in inducing fully-automated analyte transport and chaotic stirring in a straight fluidic channel embedding double-sided quarter-phase discrete electrode arrays. Our physical demonstration with multifrequency signal control on nonlinear electroosmosis provides invaluable references for innovative designs of multifunctional on-chip analytical platforms in modern microfluidic systems.

## 1. Introduction

Mixing two or multiple sample streams within a confined fluidic channel is important and challenging in the fields of chemical engineering, biomedical diagnostics, electronic cooling, and drug discovery [1]. A number of methods have been proposed over the past decade to enhance sample blending at the nanoliter scale, and can be chiefly classified into either passive or active mixer decided by the underlying fluid physics [2,3]. Since passive mixing extends the contact phase interface, increases the diffusion time, and destabilizes the laminar flow mode between co-flowing liquid media driven by an external pressure gradient, by inserting complex curved geometry or solid barriers into the internal channel, it depends entirely on molecular diffusion or hydrodynamic chaotic advection. [4]. On the other hand, active microfluidic mixers exploit external energy input to initiate fluid motion for improving the device mixing efficiency—e.g., acoustic/ultrasonic, magnetic, electrochemical, laser, and electrokinetic approaches [5]. In particular, electrohydrodynamic (EHD) fluid flow has been broadly applied in microsystems to realize ample mixing of fluidic samples [6,7,8,9]. The recent development of microelectronic processing technology has allowed for an ease at which closely packed microscale electrode arrays can be patterned and integrated into microfluidic channels. This opens up a new opportunity for imparting dynamic electrokinetic forces to the liquid medium by employing an external AC electric field [10,11,12,13,14,15]. In contrast with linear electroosmotic streaming on insulating charged channel sidewalls, EHD fluid motion appears as a series of flow vortex above an array of ideally polarizable metal-strip electrodes, and the voltage required is commonly no more than dozens of volts [16,17]. In this way, AC nonlinear electrokinetics is able to achieve a higher degree of freedom control on localized flow behavior, and finds interesting applications in pumping, mixing, and separation of target analyte in the context of microfluidics, in virtue of its superior flexibility by adjusting the amplitude, phase gradient, and field frequencies of the voltage wave, namely, AC electrothermal (ACET) [18,19,20,21,22,23,24,25,26,27,28,29,30,31,32,33,34,35,36,37,38] and AC electroosmosis (ACEO) [39,40,41,42,43,44,45,46,47,48,49,50,51,52,53].

ACET is originated by Maxwell-Wagner smeared structural polarization [54]. In order for the Gauss law and current continuity condition to be satisfied at the same time, charged ionic clouds are induced across a thermal gradient inside the bulk fluid submitted to external AC excitations. These induced sinusoidal charges are in turn acted by the same-frequency harmonic electric field to exert a time-averaged DEP force on the liquid body, which is proportional to the voltage squared (external heating) or fourth-power value (internal heating), and therefore steady convection of aqueous solution can be expected even in oscillating fields [55]. In the latter, since the electrothermal body force increases linearly with the liquid ionic strength to the leading order, ACET is effective in driving high-conductivity biofluids within the range of 0.05–5 S/m. However, for dilute electrolyte with conductivity no more than 0.01 S/m, ACET will lose effect, unless artificial heating elements are delicately embedded into the insulating substrate, while doing this would need intricate micromachining steps.

Unlike ACET that causes electroconvection in the bulk phase of concentrated solutions, ACEO is a kind of surface streaming flow, in which the electrostatic interaction of the external AC voltage wave with its own induced charge cloud within an induced double layer (IDL) drives a net electrokinetic slipping flow right outside the Debye layer [56,57,58]. Since it relies mainly on electrochemical polarization at the electrode/electrolyte interface, ACEO serves as a method of choice for actuating fluid flows of dilute electrolyte, wherein ion overscreening rather than overcrowding dominates for low ion-conductivity environment [59,60]. It has been reported that both standing-wave (SW) and traveling-wave (TW) AC voltage signals can be utilized for producing ACEO streaming flows in microchannel embedding metal-strip electrode arrays. SW-induced ACEO usually generates vortex fluid motion adjacent to the oppositely-polarized electrode pair, with great potential in causing analyte mixing [61]. On the other hand, TW-induced ACEO (TWEO) can induce parallel streamlines above a linear electrode track of continuous phase transition in sinusoidal voltage at the double-layer dispersion frequency [62,63,64,65,66,67]. To the best of our knowledge, however, ACEO and TWEO have never been applied together in the same fluidic device before, and the scientific information of their combined effect on the resultant microflow behavior is still missing as well.

**Figure 1:**
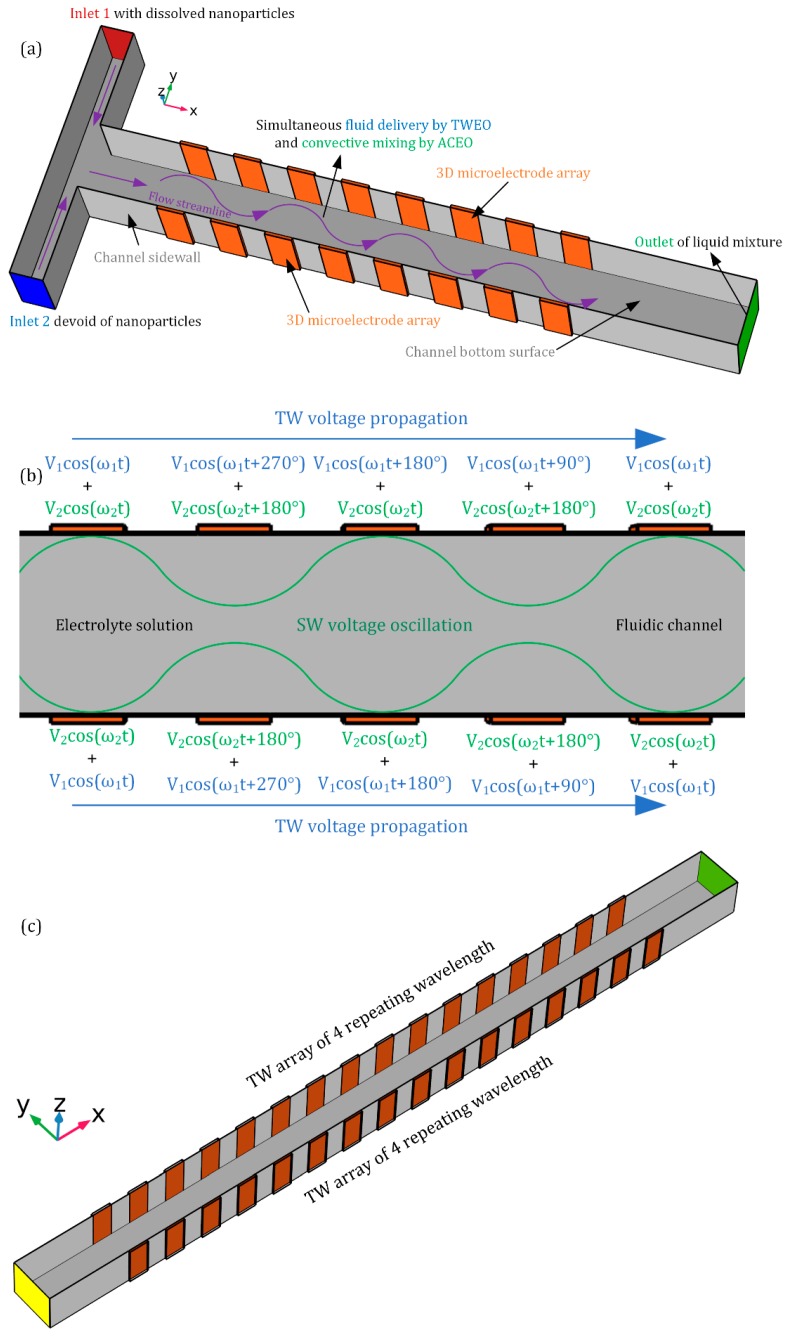
3D schematic diagram of MICEO-enabled pump-free liquid transport and mixing in a microfluidic device embedding double-sided 3D metal-strip electrode arrays. For a proof of concept study, a discrete quarter-phase *TW* voltage signal of amplitude *V*_1_ (*V_TW_*) and field frequency *f*_1_(*f_TW_*), as well as a *SW* potential gradient of amplitude *V*_2_ (*V_SW_*) and oscillating frequency *f*_2_ (*f_SW_*) are first superimposed and then applied to the sidewall discrete electrode arrangement. During exposure to the dual-frequency phase-shifted electric fields, any micro/nano-scale solid entities within the working fluid can be delivered unidirectionally by TWEO and stirred by transverse ACEO vortex flow field at the same time. (**a**) In this way, we can realize simultaneous pumping and convective mixing of chemical analytes in a straight microchannel by introducing the fluid physics of MICEO. (**b**) Detailed information on the voltage signals imposed on the sidewall electrode arrays. A progressively phase-shifted traveling potential wave of four discrete phases oscillates at frequency ω_1_ and propagates toward the downstream outlet port, which is then effectively added to a standing-wave voltage gradient at frequency ω_2_. The synthetic electrical signal is ultimately imposed to the consecutively-distributed 3D sidewall electrode strips for inducing helical streamlines that enable the device dual role in pumping and mixing at the same time. (**c**) The full-scale 3D computational domain applied in the FEM-based commercial software package (Comsol Multiphysics 5.3a).

To address the above issue, we present herein a unique concept of multifrequency nonlinear induced-charge electroosmotic (MICEO) streaming, in terms of a brand-new manipulation tool of particle and liquid contents in microfluidic systems. As shown in Figure 1, two types of AC signals of distinct field frequencies—i.e., one standing and one traveling potential wave—are first added together and then imposed to double-sided discrete electrode array arranged on either side of the channel sidewalls. A mathematical analysis considering dual Fourier-mode actuation indicates that, the global feature of the electrokinetic flow streamlines can be flexibly adjusted by making a change in the voltage amplitudes and exciting frequencies of the hybrid SW/TW signals. The synthetic flow field pertinent to MICEO can be made to either have greater vorticity or produce faster unidirectional pump flow rate, as ACEO or TWEO of distinct actuating frequencies dominates the resultant fluid physics, respectively. Then, the effectiveness of this physical phenomenon is validated by particle tracing experiment above a confocal spiral microelectrode array using a special powering technique. Finally, by conducting direct numerical simulation, we visually clarified that MICEO in the presence of both in-phase and out-of-phase electrode polarizations can spawn a series of EHD micro-vortices in the lateral direction and ICEO fluid transport along the axial direction, which is in favor of realizing simultaneous unidirectional delivery and convective mixing of nanoparticles even without the need of the application of an external pressure gradient. These results offer precious physical insight into developing flexible microfluidic platforms for fully-automated sample treatment with MICEO.

## 2. Methods

### 2.1. Geometry Configuration of the MICEO-Enabled Microfluidic Device for Simulations Study 

In this work, we first conducted a theoretical and simulation analysis on the flow pattern of MICEO adjacent to two parallel traveling-wave electrode arrays of four discrete phases, which are arranged on the opposite sides of the fluidic channel having a vertical height of *H_c_*, as shown in Figure 1. The basic device geometry for enabling MICEO streaming next to the ideally polarizable phase-shifted metal strips is displayed in Figure 1a, wherein the central canal of width *W_c_* and length *L_c_* is sandwiched by two linear *TW* electrode tracks of *n* = 4 repeating wavelength on both sides.

Two AC potential waves of distinct oscillating frequencies are applied to the multiphase discrete electrode arrangement at the same time. Firstly, a progressively phase-shifted *TW* voltage signal with four discrete phases is imposed to the two electrode strip layers of nanometer thickness in sequence, which are mounted on both channel sidewalls along the channel length direction. A discrete traveling-voltage wave of an explicit form, $\phi_{1}^{i}=V_{1}\cos(\omega_{1}t-2\pi (i-1)/4)$, where *i* = 1, 2, 3, 4 denotes the *i*-th electrode, moves along the level of linear electrode track towards the channel exit. Since both electrode width and separation have an identical size W_E_, the *TW* voltage has a wavelength of λ = 8W_E_ that reflects the spatial periodicity of the Fourier mode causing out-of-phase electrochemical polarization. Secondly, we apply an in-phase *SW* potential gradient to the adjacent oppositely-polarized electrodes, i.e., $\phi_{2}^{i}=V_{2}\cos(\omega_{2}t-2\pi (i-1)/2)$, where *i* = 1, 2 stands for the *i*-th electrode in every repeating wavelength. It is noteworthy that *TW* and *SW* electric fields have distinct driving frequencies (ω_1_ and ω_2_, respectively), so that the hybrid sinusoidal voltage signal has a dual Fourier-mode nature, as shown in Figure 1b.

While ACEO fluid motion produced by the *SW* electric field gives rise to series of chaotic micro-vortices along the channel width direction, TWEO due to the traveling potential wave applied along the level of the discrete electrode arrangement produces a quite constant pump flow component along the length direction. Consequently, these two kinds of nonlinear electroosmotic flow fields, both of which intersect perpendicularly with one another, may coact and produce helical streamlines that are able to continuously transport and stir the incoming fluidic samples, even without an external pressure difference, as shown in Figure 1a. 

### 2.2. Theory and Mathematical Model of Multi-Frequency ICEO

In ACEO, the normal potential gradient is responsible for charging IDL capacitance, which is subsequently driven by the tangential counterpart into electroconvection. So, ICEO fluid motion has a quadratic dependence on the imposed voltage, and therefore it is still valid under low-frequency AC forcing. In the scientific history, Ramos and co-workers [56] conducted the most pioneering investigations of induced double layer (IDL) charging on coplanar electrodes subjected to a low-frequency harmonic actuation, wherein the initial normal field component is provided by the free surface charge induced on conducting surfaces, and unveiled that the electric field right outside the diffuse screening cloud supplied by the metal strips can act on its own induced counterionic charge layer at the ideally polarizable surfaces of block electrodes, so as to generate ACEO under field frequencies on the order of the inverse *RC* time constant for electrochemical ion relaxation. Since the Debye screening charge appears as a consequence of the externally-imposed voltage gradient, both the induced surface charge and tangential AC forcing are sinusoidal functions of the observation time, resulting in net electrokinetic streaming, even in low-frequency alternating fields.

Following the pioneering work of Ramos and co-workers [57,58] on ACEO in microchannels embedding coplanar electrode strips, Bazant and Squires [43,44] argued that an externally-applied background electric field can push forward its own induced counterion charge within the diffuse double layer on a polarizable solid surface immersed in electrolytes. In this way, a nonlinear Coulomb force is exerted inside the thin Debye layer, and they make use of the academic term ‘induced-charge electroosmosis (ICEO)’ to vividly illustrate the interesting phenomenon of nonlinear electroosmotic streaming on solid surfaces of finite electrical properties. ICEO conceptually includes ACEO/TWEO on electrode arrays subjected to external wiring, and ICEO on floating leaky dielectric solid surfaces, as well as electroconvective instability (EI) near a ion-selective medium [44].

In current analysis, since each individual metal strip in the double-sided electrode array has an explicit AC potential pre-set by a multiphase sinewave generator, we choose to use the term ACEO or TWEO to indicate that the nonlinear electroosmosis is directly originated from the surface of driving electrodes and there are no floating electrodes in the device channel.

The hybrid TW/SW electrical signal contains two Fourier modes of distinct field frequencies, i.e., *f*_1_ = *f*_tw_ and *f*_2_ = *f*_sw_. In fact, it is reasonable to solve for the *TW* and *SW* fields separately, in analogy to the mathematical treatment of multifrequency DEP [68]. A commercial software package, COMSOL Multiphysics (version 5.3a) is used to numerically obtain the flow field of TWEO, ACEO, as well as their combined effect on sample treatment. 

Our theoretical analysis hires the linear *RC* circuit theory which is valid under the Debye-Hückel limit. Therefore, we can safely divide the microsystem into two contiguous regions, including the IDL on the electrode surface and bulk phase of the liquid solution. Within the latter, to the leading order, the ion concentration is homogenous, so the electric current conservation is safely reduced to the Laplace equation for both the *TW* and *SW* fields [6]

(1a) $$\nabla^{2}{\tilde{\phi }}_{1}=0\;(\mathrm{for}\;TW\;\mathrm{field}\;\mathrm{component})$$

(1b) $$\nabla^{2}{\tilde{\phi }}_{2}=0\;(\mathrm{for}\;SW\;\mathrm{field}\;\mathrm{component})$$

In above equations, we have invoked complex variable analysis under sinusoidal excitations, that is, ϕ1(t)=Re(ϕ˜1ejω1t) and ϕ2(t)=Re(ϕ˜2ejω2t). The IDL at the ideally polarizable interface is in effect a series capacitance of a Stern layer *C_S_*, and a diffuse layer $C_{D}=\varepsilon /\lambda_{D}$. Here, $\lambda_{D}$ denotes Debye length, and ε the solution permittivity. The combined capacity has a value of $C_{0}=C_{D}C_{S}/\left(C_{D}+C_{S}\right)=C_{D}/\left(1+\delta \right)$, in terms of the surface impedance ratio $\delta =C_{D}/C_{s}$. Counterion screening inside the liquid phase enters into consideration, so to account for the Coulomb force within the IDL. Accordingly, the Ohm current from the bulk has to relay the displacement current flowing across the thin boundary layer, in terms of a current continuity on the surface of those metal strips [69,70,71,72]

(2a) $$\sigma n\cdot \nabla {\tilde{\phi }}_{1}=j\omega_{1}\frac{C_{D}}{1+\delta }\left({\tilde{\phi }}_{1}-{\tilde{\phi }}_{1}^{i}\right)\;\mathrm{for}\;\mathrm{the}\;i\mathrm{th}\;\mathrm{terminal}\;\mathrm{in}\;\mathrm{the}\;TW\;\mathrm{field}$$

(2b) $$\sigma n\cdot \nabla {\tilde{\phi }}_{2}=j\omega_{2}\frac{C_{D}}{1+\delta }\left({\tilde{\phi }}_{2}-{\tilde{\phi }}_{2}^{i}\right)\;\mathrm{for}\;\mathrm{the}\;i\mathrm{th}\;\mathrm{terminal}\;\mathrm{in}\;\mathrm{the}\;SW\;\mathrm{field}$$

where σ is the liquid conductivity, ${\tilde{\phi }}_{1}$/${\tilde{\phi }}_{2}$ the TW/SW potential at the IDL’s outer rim. ${\tilde{\phi }}_{1}^{i}$/${\tilde{\phi }}_{2}^{i}$ denotes the TW/SW body potential of the *i-*th sidewall electrode in each repeating wavelength, and n the unit vector normal to the conducting surface. We can get a characteristic *RC* dispersion frequency $f_{RC}=\left(1+\delta \right)\sigma \lambda_{D}/2\pi \varepsilon W_{E}{=O(10}^{2∼3})\;\mathrm{Hz}$ in the context of dilute electrolyte according to Equation (2). 

The zeta potential phasor induced by the TW/SW signals, as well as the natural counterpart across the diffuse screening cloud are respectively given by

(3a) $${\tilde{\zeta }}_{1}=\frac{1}{1+\delta }\left({\tilde{\phi }}_{i}^{i}-{\tilde{\phi }}_{1}\right)\;(\mathrm{zeta}\;\mathrm{potential}\;\mathrm{for}\;TW\;\mathrm{field})$$

(3b) $${\tilde{\zeta }}_{2}=\frac{1}{1+\delta }\left({\tilde{\phi }}_{2}^{i}-{\tilde{\phi }}_{2}\right)\;(\mathrm{zeta}\;\mathrm{potential}\;\mathrm{for}\;SW\;\mathrm{field})$$

(3c) $$\zeta_{\mathrm{fixed}}=\frac{\sigma_{\mathrm{free}}\lambda_{D}}{\varepsilon }\;(\mathrm{natural}\;\mathrm{zeta}\;\mathrm{potential})$$

where $\sigma_{\mathrm{free}}$ denotes the fixed charge density on insulating charged surfaces due to chemical adsorption. Since the discrete electrode array is physically embedded on channel sidewalls, Coulomb force within the electrical double layer overwhelms any other electrokinetics, resulting in a hybrid ICEO slip flow at the solid/electrolyte interface

(4) uslip(t)=−εη(ζfixed+Re(ζ˜1ejω1t)+Re(ζ˜2ejω2t))(Re(E˜1tejω1t)+Re(E˜2tejω2t))=−εη[ζfixed(Re(E˜1tejω1t)+Re(E˜2tejω2t))+Re(ζ˜1ejω1t)Re(E˜1tejω1t)+Re(ζ˜1ejω1t)Re(E˜2tejω2t)+Re(ζ˜2ejω2t)Re(E˜1tejω1t)+Re(ζ˜2ejω2t)Re(E˜2tejω2t)]

With unequal driving frequencies (*f*_1_ ≠ *f*_2_), the time-averaged slipping velocity resulted from the hybrid TW/SW voltage signal is given by

(5) 〈uslip〉=−ε2ηRe(ζ˜1E˜1t*)+−ε2ηRe(ζ˜2E˜2t*)

where the asterisk ^*^ denotes the complex conjugate, <…> the time-average operator, the superscript *t* the tangential electric field component. From preceding equation, we have made it clear that the combined MICEO liquid motion is in effect a direct linear superposition of the TWEO and ACEO flow field, as long as the Reynolds number is negligibly small. 

As for incompressible Newtonian fluid, the fully-developed velocity field abides by the Stokes equation [73]

(6a) $$-\nabla p+\eta \nabla^{2}u=0$$

(6b) ∇⋅u=0

where η represents the dynamic viscosity, and *p* the static pressure of the liquid medium. Equation (6) is subjected to MICEO slipping $\langle u_{S}\rangle$ on the double-sided multiphase electrode arrays.

A standard convection–diffusion equation is utilized to calculate the concentration distribution of incoming analytes in the fluidic channel, which arises from a combined action of molecular diffusion effect and electroconvection due to MICEO slipping [74,75,76]

(7) $$\nabla \cdot \left(uc-D_{\mathrm{solute}}\nabla c\right)=0$$

where *c* is the concentration field of nanoparticles dispersed in the liquid medium, and *D*_solute_ = 10^−11^ m^2^/s their diffusivity in a concentration gradient, which is determined from Einstein equation for solid nanospheres with 40 nm in diameter.

### 2.3. Numerical Simulation

We employ a FEM-based commertial software package, Comsol Multiphysics 5.3a, to make analysis of the induced charge electrokinetic flow next to the metal electrode arrays and its practical application in fully-autmomated sample treatment in microfluidics, with the full-scale 3D computational domain shown in Figure 1c. Considering an actual operation in realistic conditions, a double-sided electrode strip array with a finite number *n* = 4 of the repeating wavelength is used in the simulation work (Figure 1c). 

The calculation procedure of MICEO and electroconvection-enabled multidirectional mass transfer in the fluidic channel is as follows. At first, we calculate the Laplace equation Equations (1a) and (1b) for the complex amplitude of *TW* and *SW* voltage, with the electrode surface subjected to the *RC* charging boundary condition Equations (2a) and (2b), respectively. In terms of potential phasor, the *TW* and *SW* body potential of the driving electrodes are ${\tilde{\phi }}_{1}^{i}=V_{1}e^{-j2\pi \left(i-1\right)/4}$ (*i* = 1,2,3,4) and ${\tilde{\phi }}_{2}^{i}=V_{2}e^{-j2\pi \left(i-1\right)/2}$ (*i* = 1,2) respectively for Equations (2a) and (2b). Normal component of total electric current density $n\cdot \nabla \tilde{\phi }=0$ vanishes at other insulating surfaces to close the electrical boundary-value issue. Then, once both the *TW* and *SW* electric field components are known, they are substituted into Equation (5) to calculate the time-averaged MICEO slipping flow. Subsequently, the EHD fluid motion in the bulk Equation (6) is computed by imparting Equation (5) to these ideally polarizable surfaces embedded on both channel sidewalls. Both inlet and outlet are set as open boundarys. Besides, non-slip and no penetration n⋅u=t⋅u=0 are imposed on all the other insulating channel sidewalls as well as the chamber top and bottom surface. Finally, transportation equation Equation (7) is computed for obtaining the analyte concentration distribution inside the fluidic channel under the combined action of diffusive and electroconvective mass transfer. Any normal flux is forbidden on the electrode and channel inner surfaces. Fluorescent particles of *r* = 20 nm in radius with a thermal diffusivity of *D* = 10^−11^ m^2^·s^−1^ are employed in the simulation analysis [77,78,79], the concentration of which is set to *c* = 1 mol·m^−3^ at the left side and 0 mol·m^−3^ at the right side of the upstream entrance, respectively. In addition, the normal diffusion flux disappears at the outlet plane, so as to reconstruct the actual situation for sample treatment in microfluidics. 

We make use of stationary solvers for the set of control equations submitted to boundary conditions from physical constraints. The complex SW/TW potential phasor, MICEO fluid motion and sample delivery are separately solved in sequence. Free tetrahedral meshes are employed for discretization of the full-scale 3D computational geometry used in the numerical simulation (Figure 1c), and the maximum grid size near the electrode edges is assigned to be no more than 1/10 (2 μm) of the span of an individual metal strip, with a grid growth factor of 1.05 as the meshes extend from the conducting surface to the leaky dielectric bulk suspension. During the numerical computation, we preferentially apply the PARDISO solver because of its quicker iteration speed. The Reynolds number in current situation is no more than one, i.e., Rey = ρfuWe/η~O(0.02) < 1 for *u* = 1 mm/s and W_e_ = 20 μm, so that MICEO slipping flow is laminar, not turbulent in essence. Even so, under externally-imposed dual-Fourier-mode AC forcing, MICEO slip can still engender time-averaged axial pump flow and transverse vortex streaming in the bulk phase, due to its quadratic voltage dependence no matter how many actuating frequencies are engaged, which results in its dual functionality in simultaneous sample delivery and convective mixing in microscale ducts.

## 3. Results and Discussion

### 3.1. Characterization of the ACEO Flow Component

For analytical convenience, geometry size of the device design keeps unchanged during the most preliminary study: *W_e_* = *W_ee_* = 20 μm, *n* = 4, *Lc* = 0.8 mm, *Wc* = 50 μm and *Hc* = 40 μm, i.e., with an appropriate cross-sectional aspect ratio of 1.25. The PDMS channel walls on both sides sandwich a central straight duct full of conducting electrolyte solution. The test values for various physicochemical properties are as follows: *σ* = 0.001 S/m, *ε* = 80 ε_0_, *C_s_* = 0.8F/m^2^, *λD* = 37.6 nm, *δ* = 0.0235, η = 0.001 Pa·s, *c*_0_ = 1 mM, $f_{RC}=\left(1+\delta \right)\sigma \lambda_{D}/2\pi \varepsilon W_{E}$ = 432.8 Hz, and *λ* = 8W_e_ = 160 μm. Since *λD*/*λ* = 2.35 × 10^−4^ < 0.001, it is reasonable to arouse the thin layer approximation in this situation. 

Due to a dynamic force balance between Coulomb attraction and thermal diffusion at a solid/saline-solution interface, a Debye screening layer, inside which counterion charge cloud overwhelms the coion counterpart, would develop naturally due to the fixed free charge density chemically adsorbed on the surface of a solid object immersed in electrolytes, or compulsively under externally-imposed electric fields. In this work, since there is no DC voltage applied to the driving electrode array, electrostatic force within the native EDL always time-averages to zero under multifrequency AC forcing. For this reason, we mainly concentrate on the latter effect of field-induced diffuse charge dynamics, that is, capacitive charging of the IDL on ideally polarizable surfaces of the blocking electrodes caused by the hybrid sinusoidal SW/TW signals imposed sequentially on the consecutively-distributed metal strips.

**Figure 2:**
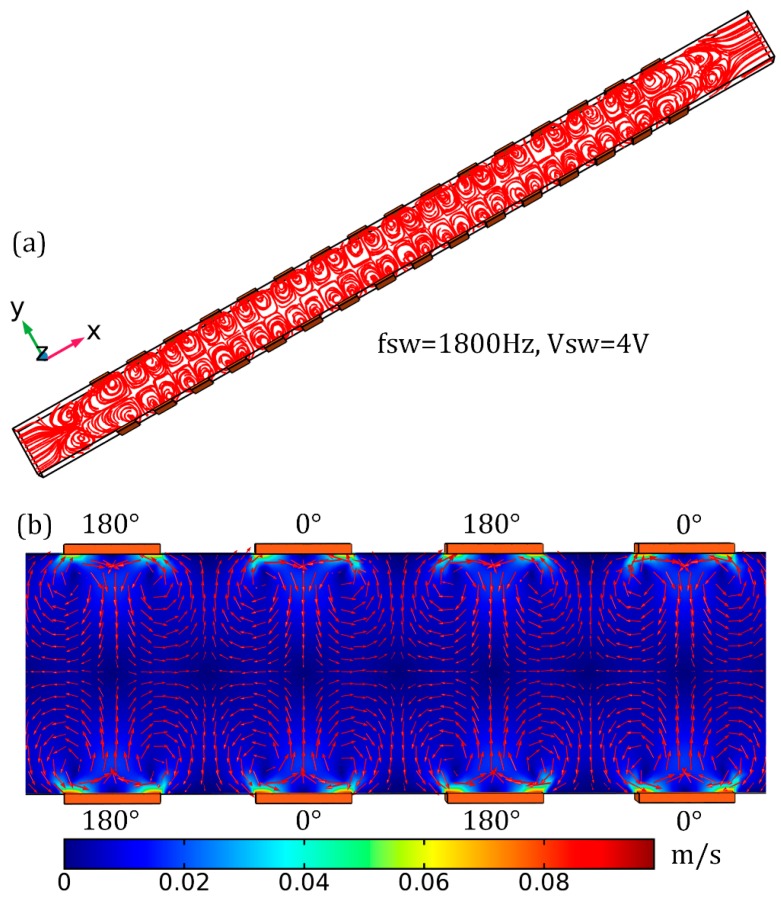
Computational visualization of the ACEO fluid motion due to the individual application of the *SW* signal voltage, namely, *V_TW_* = 0 V. (**a**) ACEO streamline distribution in the 3D microfluidic device embedding double-sided four-phase discrete electrode array of *n* = 4 repeating wavelength, and (**b**) A surface and arrow plot of ACEO vortex flow field at *V_SW_* = 4 V, *f_SW_* = 1800 Hz in the horizontal central plane of the fluidic chamber with *z* = 20 μm.

At the very start, we pay attention to the flow pattern due to the separate action of ACEO and TWEO above the phase-shifted 3D sidewall electrodes. First and foremost, when only *SW* signals are supplied to the microsystem (*V*_1_ ≠ 0 and *V*_2_ = 0), although TWEO is absent, a series of chaotic EHD micro-vortices are induced throughout the channel by ACEO (Figure 2).

In DC limit, considering rather complete Debye screening effect, most of the applied *SW* voltage drops across the diffuse screening cloud formed at the electrode/suspension interface, resulting in negligibly small electric field strength in the bulk phase. As the actuating frequency of the *SW* voltage grows and exceeds the reciprocal *RC* time scale $f_{RC}$ for the equivalent circuit of the double-layer impedance in series connection with the bulk resistance, incomplete charge screening occurs on the electrode surface when considering electrochemical relaxation within the thin boundary layer, resulting in evident leakage of electric field lines into the liquid suspension.

**Figure 3:**
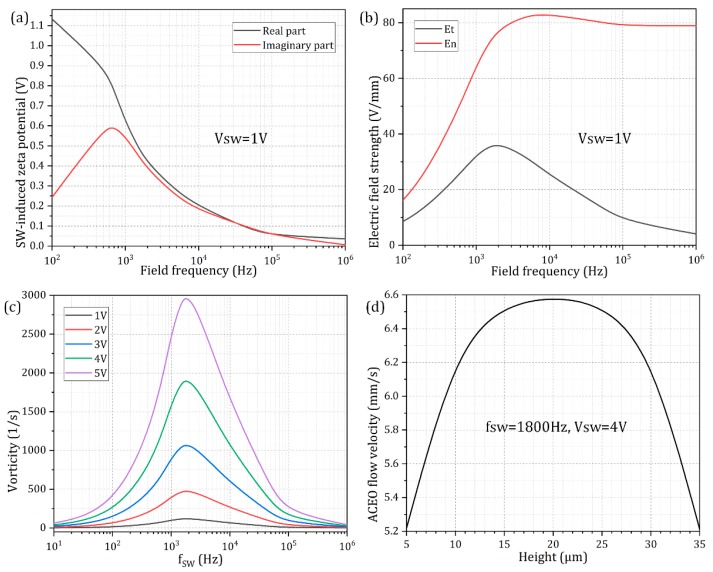
Quantitative calculation results of the electric field (**a**,**b**) and flow field (**c**,**d**) information in the situation of ACEO. (a,b), for a given voltage amplitude *V_SW_* = 1 V, (a) real and imaginary parts of the zeta potential induced by the applied *SW* voltage signals as a function of the imposed frequency, (b) Frequency dependence of the tangential and normal components of the electric field vector right outside the IDL on those conducting surfaces. (c) Frequency dependence of hydrodynamic vorticity magnitude from ACEO for a series of *SW* voltage amplitude, wherein the ideal driving frequency that holds potential for convective mixing has always an identical value around *f* = 1800 Hz; (d) ACEO flow velocity as a function of vertical distance from the channel bottom surface at *fsw* = 1800 Hz and *Vsw* = 4 V.

In this way, the potential drop between the metal surface and electrolyte bulk drops rapidly for frequencies beyond f ≈ 700 Hz (the black line in Figure 3a), and the enhanced electric field intensity caused by this factor (Figure 3b) helps push forward the counterions accumulated within the IDL more fiercely, giving rise to a single relaxation peak of out-of-phase Debye screening charge on each electrode surface around *f* = 700 Hz (the red line in Figure 3a). As the field frequency further increases and even exceeds the reciprocal *RC* time scale f ≈ 700 Hz, however, electrochemical ion relaxation takes place on those metal surfaces, so that both the real and imaginary components of the induced zeta potential from ACEO would decay at the electrode/electrolyte interface. 

Accordingly, as we raise the electric field frequency of the *SW* voltage signal from DC limit to 1 MHz, the in-phase double-layer voltage drop decreases monotonously due to a relaxation process. On the contrary, however, the out-of-phase counterpart is maximized within an intermediate frequency range around *f* = 700 Hz and manifests as a bell-shaped curve (the red line in Figure 3a), implying ACEO flow velocity would be appreciably suppressed once the signal frequency further keeps away from the key frequency. At low signal frequencies, all of the applied *SW* voltage drops across the IDL above the double-sided electrode array, leaving no electric field in the bulk (the red line in Figure 3b). Under the high frequency limit, relaxation dynamics starts to become apparent, namely, there is insufficient time for the induced space charge to accumulate within the diffuse double layer on the surface of blocking electrode in each half cycle of the AC voltage wave, giving rise to negligibly small tangential field component (the black line in Figure 3b). Therefore, double-layer charging is most evident around the critical frequency for ACEO, and any aberration of imposed field frequency from this key point would attenuate the ACEO phenomenon.

As the frequency rises, the normal electric field component right outside the IDL would increases gradually as well (the red line in Figure 3b), while its tangential counterpart varies non-monotonically and reaches a single peak value at an intermediate frequency (the black line in Figure 3b). Since the electrode bars would recover from an insulator in low-frequency limit to its intrinsic role of an ideal conductor (Figure 3b) within high-frequency range with a characteristic microscopic distance scale O(*λD*) off these ideally polarizable surfaces, it behaves more as a typical leaky dielectric around the *RC* dispersion frequency where the tangential part of the electric field lines maximizes. 

Besides the effect of induced double-layer charging, another indirect consequence of electrochemical polarization is the induction of fluid motion due to ICEO slipping at low signal frequencies. When employing a *SW* voltage wave, ICEO manifests as a series of counter-rotating micro-eddies near the electrode array, namely, ACEO. As shown in Figure 2a,b, for the full-scale 3D computational space where a double-sided discrete electrode array of four repeating wavelengths is integrated into both sidewalls of a straight fluidic channel, since ACEO is originated by nonlinear Coulomb force inside the thin IDL, the time-averaged ACEO flow velocity is quickest right outside the Debye layer (in coincidence with the electrode surface in the simulation), and vanishes in the midchannel located in between the opposing electrode array due to viscous diffusion of liquid momentum (Figure 2b). From the linear asymptotic analysis by Gonzalez et al. In [57], ACEO is only significant around *RC* relaxation frequency, and vanishes in both low (due to complete Debye screening) and high (due to electrochemical ion relaxation) frequency limit. This can be well validated by Figure 3c, in which the vorticity magnitude due to ACEO attains a single relaxation peak at the inverse *RC* time constant for capacitive charging of electrical double layer $f_{RC}^{SW}$ = 1.8 kHz, which is about two orders of magnitude smaller than the Debye relaxation frequency $f_{\mathrm{MW}}=\sigma_{f}/{2\pi \varepsilon }_{f}=225\;\mathrm{kHz}$ of the bulk fluid. In the analytical model of linear *RC* circuit theory, the characteristic inverse *RC* time scale for charging of the induced double layer on ideally polarizable surfaces is given by $f_{RC}=\left(1+\delta \right)\sigma \lambda_{D}/2\pi \varepsilon R$, which should be in accordance with the simulation result 1.8 KHz from the bi-layer mathematical model used in current work. Here, *δ* denotes the surface capacitance ratio, *σ* the liquid conductivity, *λD* the Debye screening length, R the characteristic microscopic length scale for ACEO. In this sense and in order for the theoretical prediction to match the simulation results, *R* ought to equal approximately one-fourth of the electrode width, i.e., *R* = W_e_/4 = 5 μm. 

Since a full-scale 3D numerical model has been adopted in our simulation analysis, the ACEO flow velocity also makes a change with a variation in the suspension height of the particle sample (Figure 3d). As shown in Figure 3d, the surface-averaged electrokinetic flow rate attains a peak value at the middle horizonal section of the fluidic chamber, while it decays sharply once the place of interest further approaches the channel top and/or bottom surface, as caused by an adverse influence of the presence of viscous boundary layer at the boundary of the simulation domain.

### 3.2. TWEO Pump Behavior Driven by A Single Phase-Shifted Harmonic Field

**Figure 4:**
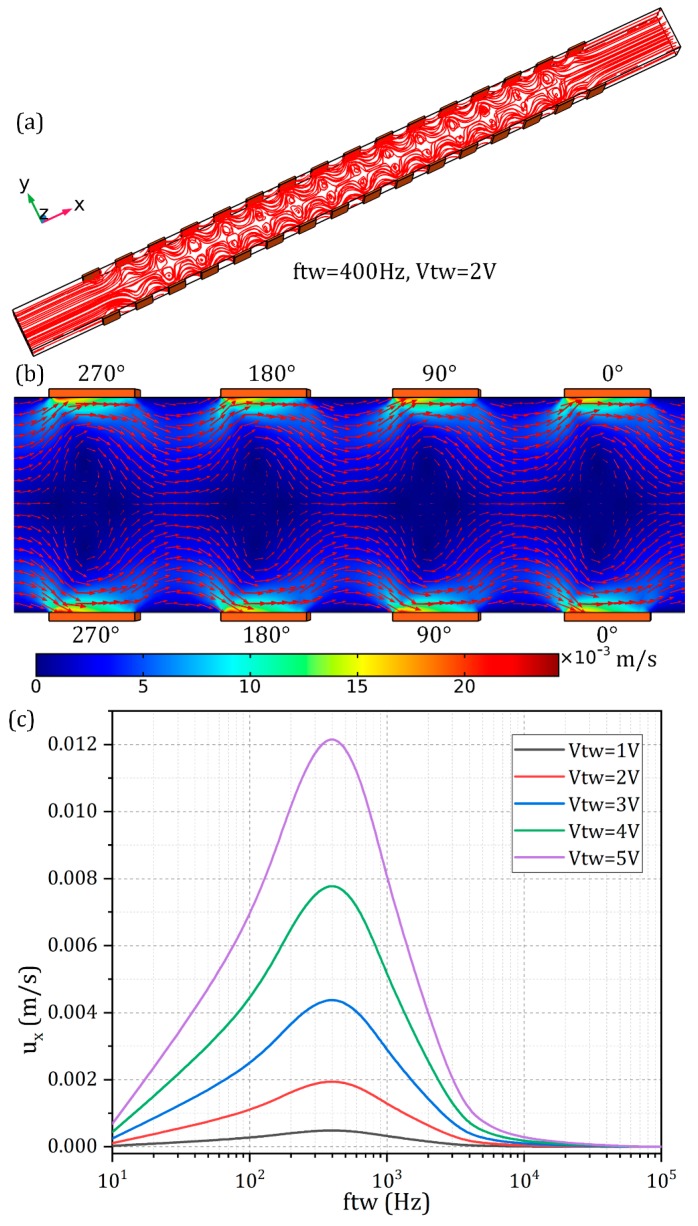
Numerical investigation of TWEO fluid motion caused by a pair of progressively phase-shifted *TW* potential signals synchronously moving along the top and bottom rows of the double-sided electrode arrays. (**a**) TWEO pumping streamline distribution in the 3D microfluidic device with a channel height of *Hc* = 40 μm, and (**b**) a surface and arrow plot of TWEO pump flow field at *V_TW_* = 2 V and *f_TW_* = 400 Hz in the horizontal central plane of the fluidic chamber, with the most salient feature of TW-induced co-field unidirectional fluid transport; (**c**) frequency dependence of the averaged pump flow rate in the direction of the traveling wave within the range of *TW* voltage amplitude from *V_TW_* = 1 V to 5 V.

We then arbitrarily remove ACEO by constraining *V*_SW_ = 0 and focus on the effect that an externally-imposed traveling potential wave may have on the resultant ICEO flow field, that is, TWEO. Pumping of electrolytes by TWEO arises from the action of an applied *TW* electric field on its own induced Debye screening charge within the thin IDL, which is formed on top of the ideally polarizable surface immersed in saline solution. In this study, we realize such a traveling field via the same double-sided discrete electrode array as used in previous simulation analysis for ACEO convection (Figure 4a), upon which the corresponding sinusoidal voltages of correct phase sequence are imposed. 

Unidirectional movement of liquid element is observable on application of a traveling wave potential to the double-sided electrode structure at frequencies on the order of the inverse *RC* time constant of the saline solution. On imposing a *TW* signal propagating downstream, the fluid motion is directed straightly to the right electrolytic port, as shown in Figure 4a,b. On inversing the travel direction of the sinewave, the parallel laminar streamlines reverse direction and move from the right to the left side (not shown). Thereby, it is definite that the traveling wave potential is responsible for the phenomenon of directed liquid transport. 

As shown in Figure 4c, at very low frequencies, the double-layer charge and tangential field component have a 90° phase difference and are completely out of phase, so no net flow can be produced. At the same time, the electric field in the bulk phase is weakened to great extent due to the formation of dynamic IDL in quasi-equilibrium state. As the signal frequency of the traveling potential wave approaches an intermediate characteristic relaxation frequency $f_{RC}^{TW}$ = 400 Hz, the phase lag between the Debye screening charge and tangential AC forcing decreases so that a time-averaged fluid motion in the direction of the travel of the *TW* takes place (the peak point in Figure 4c). Although the density of the induced charge cloud may decrease, the electric field right outside the Debye layer increases in strength. Since we have employed herein the linearization theory, the ideal operating frequency of the electroosmotic pump is independent of the *TW* voltage amplitude (Figure 4c), while this would not hold true when nonlinear effects such as surface conduction and anisotropic IDL capacity have to be taken into account. With further increase of the signal frequency, the pump flow rate decays monotonously because of the occurrence of incomplete Debye screening. As a consequence, similar to ACEO, TWEO always exhibits a single relaxation peak arising from double-layer dispersion. 

Even so, ACEO and TWEO have distinct ideal manipulation frequency, which is 1800 Hz and 400 Hz in current device design, respectively. According to Equation (4), a large deviation of the two characteristic peak-flow frequencies greatly facilitates the independent control on electrokinetic fluid transport and convective mixing via separately adjusting the *TW* and *SW* voltage signals. 

### 3.3. Multi-Frequency ICEO from Combined ACEO and TWEO

In previous sections, we have demonstrated the respective role of *SW* and *TW* excitation in correspondingly producing transversal ACEO chaotic vortex and axial TWEO pump behavior. Accordingly, it is then of paramount significance to unite both ICEO phenomena and test their combined contribution to the synthetic electroosmotic flow field. For realizing MICEO, a hybrid sinewave of dual oscillating frequencies is applied to the double-sided discrete electrode array, with the specific voltage-phase sequence matching that shown in Figure 1b.

**Figure 5:**
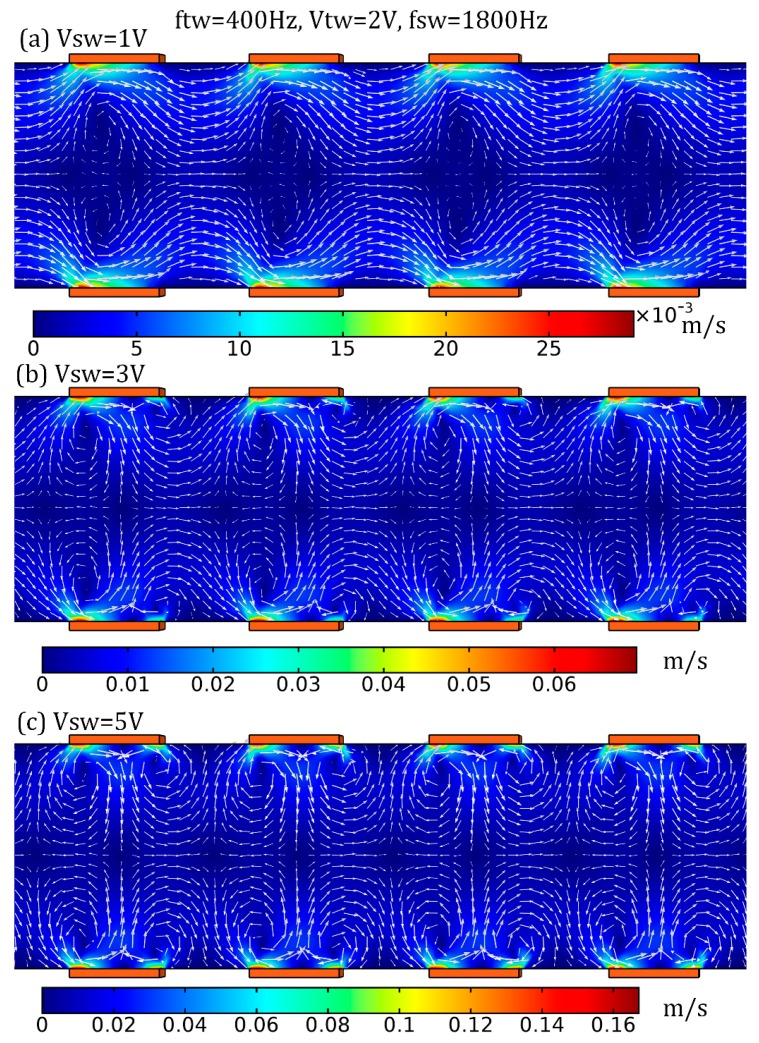
Simulation analysis of the MICEO flow behavior from combined ACEO and TWEO actuated by AC forcing of two distinct oscillating frequencies, 1800 Hz and 400 Hz, respectively, in the horizontal central plane of the 3D device design. (**a**–**c**) Impact of V_SW_ on the resultant electroosmotic flow field, as the amplitude of *TW* voltage is fixed at *V_TW_* = 2 V (unit: m/s), (a) *V_SW_* = 1 V, (b) *V_SW_* = 3 V, (c) *V_SW_* = 5 V. Accordingly, the vorticity grows sharply with the amplitude of SW signal, while the pump motion remains almost unchanged.

For analytical convenience, the *TW* voltage amplitude is fixed at *V_TW_* = 2 V, and the two voltage modes oscillate at their optimum driving frequency for enabling the most effective ACEO vortex and TWEO liquid transport in light of the flow speed, i.e., *f_SW_* = 1800 Hz and *f_TW_* = 400 Hz. As shown in Figure 5, with an increase in the *SW* voltage amplitude from 1 V (Figure 5a), 3 V (Figure 5b) to 5 V (Figure 5c), the resultant ICEO fluid motion transits from almost a pure pump mode moving in the direction of the travel of the *TW* signal to a more complicated EHD flow pattern affected simultaneously by a series of local ACEO vortices and global TWEO medium delivery towards downstream. That is, at a given *TW* field strength, increasing the *SW* signal is able to enhance the electrokinetic flow vorticity, while it plays a negligibly small effect on the pump behavior. 

**Figure 6:**
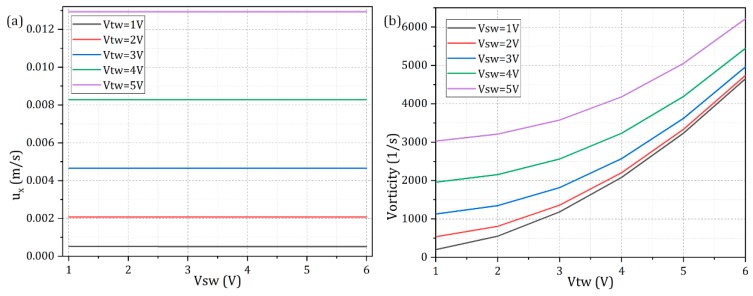
Analysis of the interplay of ACEO and TWEO with respect to the MICEO device performance in terms of simultaneous on-chip fluid delivery and electroconvective mixing. (**a**) *V_SW_*-dependence of the electroosmotic pump behavior (the flow speed along the channel length direction) for different *TW* voltage amplitude; (**b**) V_TW_-dependence of the resultant hydrodynamic vorticity for different *SW* potential amplitude.

Besides these qualitative pictures, flow vorticity and pump performance due to MICEO are further mathematically quantified in Figure 6. According to Figure 6a, the pump flow rate can be merely controlled by tuning the *TW* signal, and the *SW* electric field has indeed no contribution to the unidirectional fluid transport whatever the *TW* field strength is. The pump flow rate grows quadratically with the *TW* voltage, since TWEO is a nonlinear electrokinetic effect where the electric field propels its own induced charge within the IDL at the electrode/electrolyte interface. On the other hand, from Figure 6b, although it is possible for both TWEO and ACEO to adjust the magnitude of the vorticity field, *SW* signal input serves as a better method of choice for achieving separate control on device dual functionality since its variation does not exert an impact on the ultimate MICEO pump performance (Figure 6b). In this sense, the comprehensive fluid physics of MICEO can be arbitrarily reconfigured by delicately recombining a hybrid *SW*/*TW* sinewave, whose functionality can be oriented towards either convective mixing when ACEO vortex flow field dominates or fully-automated external-pump-free liquid delivery when horizontal TWEO streaming is more efficient.

### 3.4. Experimental Observation of MICEO Flow Field Above a Confocal Spiral Microelectrode Array

#### 3.4.1. Device Configuration and Experimental Method

To demonstrate the availability of current MICEO flow theory, a multiphase electrokinetic microfluidic device is fabricated and then we conduct the particle tracing experiment. Multilayer fabrication process has to be applied if the configuration of a linear electrode track of different voltage phases is adopted. To avoid complicated wiring, we come up with a special device design of a confocal spiral quarter-terminal electrode array of *n* = 5 repeating wavelength, as has been used in our previous work (Figure 1 in [80]), so it is not shown here. These conducting rings are deposited on a thick glass substrate and covered by a PDMS microchannel with two opening cylindrical electrolytic ports of 2 mm in radius. The four circulating metal strips share an equal width of *L_E_* = 100 μm, and the nearest gap size between adjacent electrode phases equals *L_G_* = 30 μm. Consequently, the wavelength of single repeating spatial period is *L* = 4(*L_E_* + *L_G_*) = 520 μm, and the diameter of the whole helix is about 6 mm. One great benefit of this circulating design is that, inter-phase insulation and external wiring of the four sequential terminals can be accomplished at the periphery of the circular electrode system at a time, with much more convenience of device operation compared to that for a linear multi-phase array [67].

We choose aqueous saline solution as the potential candidate of the working fluid, its ionic conductivity is carefully controlled by modulating KCl salt and supervised via a conductivity meter. The solution conductivity for flow tracing experiment is σ = 0.001 S/m, which is in the dilute limit for actuating MICEO in the absence of ion overcrowding effect [81,82,83]. Considering the much lower ionic strength of the bulk fluid, the ITO metal strips can be treated as ideally polarizable solid conductors in current device design. The sample particles used here were polystyrene nanospheres (Molecular Probes Co.) with diameter of *r* = 250 nm. To prepare the fluorescent nanocolloids, a 10 μL liquid solution containing 10% particle samples was diluted with the 10 μS/cm KCl solution into 2 mL, so as to keep track of the dynamic motion behavior of latex beads caused by MICEO streaming flow. The working fluid with tracer particles is then injected into the fluidic channel and eventually stuffs it.

We employ a commercial multichannel function generator (TGA12104, TTi, Cambs, UK) to generate dual-frequency sinusoidal voltage signals with controllable spatial-temporal phase transition trait. The specific transient waveform of the applied voltage is monitored using a commercial digital oscilloscope (TDS2024, Tektronix, Beaverton, OR, USA). Besides, the experimental observation window is selected preferentially at the lower left side of the ring-shaped electrode array.

#### 3.4.2. Experimental Results and Discussion

**Figure 7:**
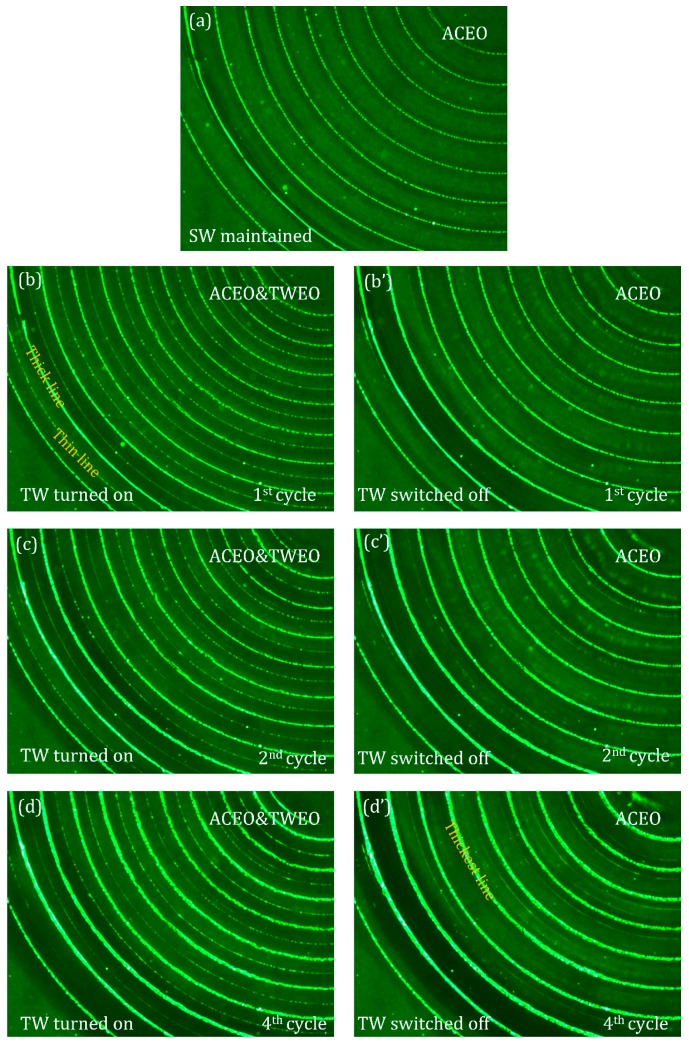
Top view of the flow tracing experiment of MICEO, which takes advantage of the confocal spiral four-phase electrokinetic microchip introduced in [80], and the observation window is chosen at the lower left side of the annular array. (**a**) At the most initial time, only a *SW* voltage signal with *Vsw* = 2 V, *fsw* = 50 Hz and 180° phase difference between every adjacent electrode is imposed to the annular array with 4 (number of discrete voltage phase) × 5 (number of repeating wavelength) metal strips, 10 particle trapping lines are formed on the annular electrode strips by ACEO vortex streaming; (**b**) after 30 s, another *TW* voltage sequence with *V_TW_* = 2 V, *f_TW_* = 70 Hz and 90° phase difference between consecutively distributed metal strips is superimposed with the already-existed *SW* signal, which generates stronger vortex flow field and an additional outward pump flow component, resulting in doubling in the number of particle assembly line, as characterized by the repeating spatial periodicity of one thin line and one neighboring thick counterpart. (**b’**) 30 s later, the traveling sinewave is withdrawn with the *SW* signal being invariably maintained, although the number of collection lines is reduced from 20 to 10, the fluorescent intensity becomes stronger indicating a better particle trapping performance. (**c**,**c’**) The second cycle of adding and removing the TWEO flow component with respect to the ACEO fluid motion that has always been maintained for a same time period of 1 min (30 s + 30 s). (**d**,**d’**) The fourth cycle of adding and deleting the TWEO pump flow component relative to the maintained ACEO for an identical time period of 1 min (30 s + 30 s). It is noteworthy that, by using the particular technique of MICEO, the particle trapping performance of ICEO is enhanced to great extent, as can be evidenced from (a,b’,c’) to (d’). Please refer to the Supplementary Video.

In essence, a hybrid phase-shifted dual-Fourier-mode sinewave is required for actuating MICEO along the electrode track in the annular device design. We introduce here a special transient powering technique, however, in order to differentiate ACEO and MICEO merely using a single experimental video (see Supplementary Video and Figure 7). At the very beginning, with the function generator switched off, the nanoparticle samples are distributed homogeneously in the electrolyte solution, and no obvious concentrating phenomenon is observable due to the domination of stochastic Brownian motion of nanoscale entities (not shown). 

On application of a *SW* voltage signal of *Vsw* = 2 V, *fsw* = 50 Hz, and 180° phase lag to the adjacently-arranged electrode strips, vortex flow field due to ACEO slipping transports the nanoparticles from the surrounding bulk medium to the centerline of the surface of the blocking electrodes, resulting in the formation of 10 particle assembly lines, as shown in Figure 7a, and it takes about 20–30 s for the dynamic trapping process to reach the steady state, when the number of particles around the flow stagnation region becomes almost saturated and cannot increase any longer. Once the *SW* signal has been maintained for 30 s and the 10 circular assembly lines have been well developed (Figure 7a), a traveling field with *V_TW_* = 2 V, *f_TW_* = 70 Hz and 90° phase difference between consecutively distributed metal strips is superimposed with the preexisted *SW* signal (Figure 7b). In this scenario, according to the theory of MICEO streaming established in preceding sections, a unidirectional pump flow component of TWEO is induced by the additional *TW* voltage signal, and it can effectively superimpose with the series of lateral ACEO micro-vortices along the channel length direction. In this sense, MICEO fluid motion from combined ACEO and TWEO is much stronger than ACEO or TWEO works alone, so more latex beads are transported and then adhered onto the ideally polarizable surfaces of the phase-shifted metal strips by the synthetic flow field of MICEO, which gives rise to an increase in the number of assembly line from 10 to 20 (Figure 7b). Very interestingly, the newly formed 10 circular trapping lines by TWEO are much thinner than that due to ACEO, so that the complete sample distribution pattern under the influence of MICEO is now characterized by the repeating spatial periodicity of one thin line from TWEO and one neighboring thick counterpart from ACEO (Figure 7b), which also takes about 20–30 s to achieve the stable state. 

As long as the MICEO-induced fluorescent lines with alternatively-placed thick and thin counterparts have been completely assembled, we switch off the *TW* voltage component immediately. With time goes on, the 10 thin trapping lines by TWEO vanish, and the sample distribution pattern recovers to the original 10 thick lines due to the action of ACEO (Figure 7b’). However, the fluorescence intensity in Figure 7b’ is made much higher than that before the application of MICEO (Figure 7a). It is our speculation that although the TWEO pumping effect no longer exists once the *TW* signal is withdrawn, the samples originally captured by TWEO would enter the actuating range of ACEO whirlpool in the bulk fluid and then be attracted onto the flow stagnation region defined by ACEO. That is, with the *TW* signal powered off while the *SW* voltage invariably maintained, the particles originally occupying these thin lines would move to their adjacent thick lines (Figure 7b,b’), resulting in further thickening of the particle trapping lines formed by ACEO, which indicates a better device performance in sample collection (Figure 7b’). 

The above experimental procedure—namely, actuating MICEO for 30 s and then remove TWEO leaving only ACEO for 30 s—is repeated four times in total, as sequentially shown in Figure 7(c,c’,d,d’). The sample assembly pattern remains identical as that in Figure 7b,b’, respectively, while the trapping performance becomes more appreciable as the repetition cycle increases, as can be clearly evidenced by a stark contrast in the fluorescent intensity between Figure 7a,d’. In this way, the existence of the physical phenomenon of MICEO streaming flow is preliminarily demonstrated by conducting particle tracing experiments, which holds great potential for flexible sample handling in modern microfluidic systems. 

### 3.5. Influence of Electrolyte Conductivity and Channel Height on MICEO Streaming

All the preceding simulations have assumed the working fluid has a quite low electric conductivity of 0.001 S/m. For the sake of biological applications, however, the buffer solution and cell culture medium usually require the liquid conductivity to be no less than 0.1 S/m, so it is necessary to test the feasibility of the actuation of an effective MICEO fluid motion at higher ion concentrations. 

**Figure 8:**
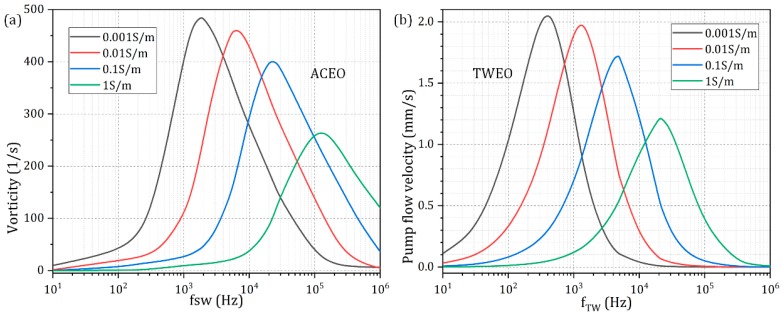
Effect of liquid electric conductivity on the resultant MICEO flow field under a finite channel height of *Hc* = 40 μm. (**a**) For *V_TW_* = 0 and *V_SW_* = 2 V, the frequency dependence of vorticity due to ACEO at varying medium conductivities. (**b**) For *V_TW_* = 2 V and *V_SW_* = 0 V, the frequency dependence of electrokinetic pump flow rate due to TWEO for different level of solution ionic strength.

For high-conductivity environment, the linear *RC* polarization model may not be so accurate as the dilute electrolyte condition, but it can still capture the most salient feature of MICEO beyond the Debye-Huckel limit. In this sense, we calculated the frequency dependence of MICEO fluid motion at four distinct level of ionic strength (Figure 8). As shown in Figure 8a, whatever the specific solution conductivity is, the flow vorticity caused by ACEO always exhibits a single double layer dispersion process at the characteristic inverse *RC* time constant of electrochemical polarization on the blocking electrodes. While the ideal flow frequency rises with the ionic strength, the peak electrokinetic flow vorticity decreases as the liquid conduction increases. Similar variation trends also hold true for the TWEO pump flow velocity (Figure 8b). That is, the optimal pump frequency enhances at higher ion concentrations, but the peak flow rate decreases sharply once the solution conductivity increases by three orders of magnitude from 0.001 S/m to 1 S/m. The reason behind is quite clear: on one hand, the rise in solution ionic strength shrinks the double-layer thickness, so that the Stern layer becomes more dominant over the diffuse screening cloud at a larger solution conductivity, which suppresses the induction process of the counterions and thereafter lowers the ICEO flow velocity and vorticity; on the other hand, a higher liquid conduction implies a smaller impedance of the bulk fluid, and accordingly, a higher characteristic relaxation frequency is needed to short-circuit the double-layer voltage drop by half and provide sufficient electric field leakage for enabling appreciable EHD fluid motion.

**Figure 9:**
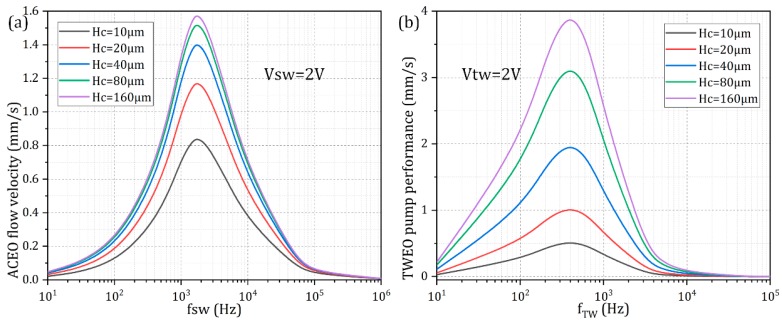
Effect of the finite channel height on the resulted MICEO fluid motion for a given liquid conductivity 0.001 S/m. (**a**) For *V_TW_* = 0 and *V_SW_* = 2 V, the frequency dependence of ACEO flow velocity for varying channel vertical dimension. (**b**) For *V_TW_* = 2 V and *V_SW_* = 0 V, the frequency dependence of TWEO pump flow rate for different channel height.

A finite channel height, which is usually encountered in practical experiment, may also exert a negative influence on MICEO fluid flows. As shown in Figure 9, although a moderate cross-sectional aspect ratio of 1.25 has been applied in preceding analysis (*Hc* = 40 μm), both ACEO fluid motion and TWEO pump behavior are very susceptible to changes in the channel vertical dimension. On one hand, as *Hc* increases from 10 μm to 160 μm, the global electroosmotic flow velocity enhances monotonously (Figure 9a,b), due to, in part, the less negative influence from the viscous boundary layers formed at the channel top and bottom wall. On the other hand, although an increase in the height of the fluidic chamber helps improve the electrokinetic flow status, the ideal operating frequency is not affected in any way (Figure 9a,b), in that it is the electrode width and separation in the horizontal plane rather than the vertical dimension that determine the characteristic polarization length scale for the actuation of ICEO. As a consequence, considering a practical application in sample manipulation with MICEO in microfluidics, it is quite feasible to raise the channel height for engendering stronger electroosmotic fluid motion and better device performance.

### 3.6. Simulation Study of Simultaneous Pumping and Convective Mixing by MICEO 

From the above analysis, MICEO is capable of achieving a high degree of freedom control on the resultant ICEO flow pattern, this excellent feature makes it convenient to use the fluid physics of MICEO for developing multifunctional fluidic devices at the micrometer dimension. As an illustrative example, we still employ the device design with *n* = 4 repeating wavelength of linear *TW* electrode track embedded along both sidewalls of a straight microchannel, as shown in Figure 1a,c. 

For the sake of analyte mixing, a T-shape junction is arranged at the upstream section, where there are a pair of face-to-face inlets for injection of two co-flowing liquid solution of identical properties. The solution passing through inlet 1 is an aqueous electrolyte containing target nanoparticles, while that entering inlet 2 is a pure saline solution of same electric conductivity and does not have any solid-state solutes. An eligible liquid mixture in high-throughput is obtainable at the channel outlet (the green plane in Figure 1a) right downstream the double-sided quarter-phase metal strip array, as long as appropriate AC voltage waves are imposed to the set of electrode pads. From the preceding analysis, however, it is appropriate for us to arbitrarily increase the height of the channel from 40 μm to 500 μm for achieving an ideal MICEO-enabled sample treatment, which is able to evidently diminish the adverse vertical channel confinement effect.

**Figure 10:**
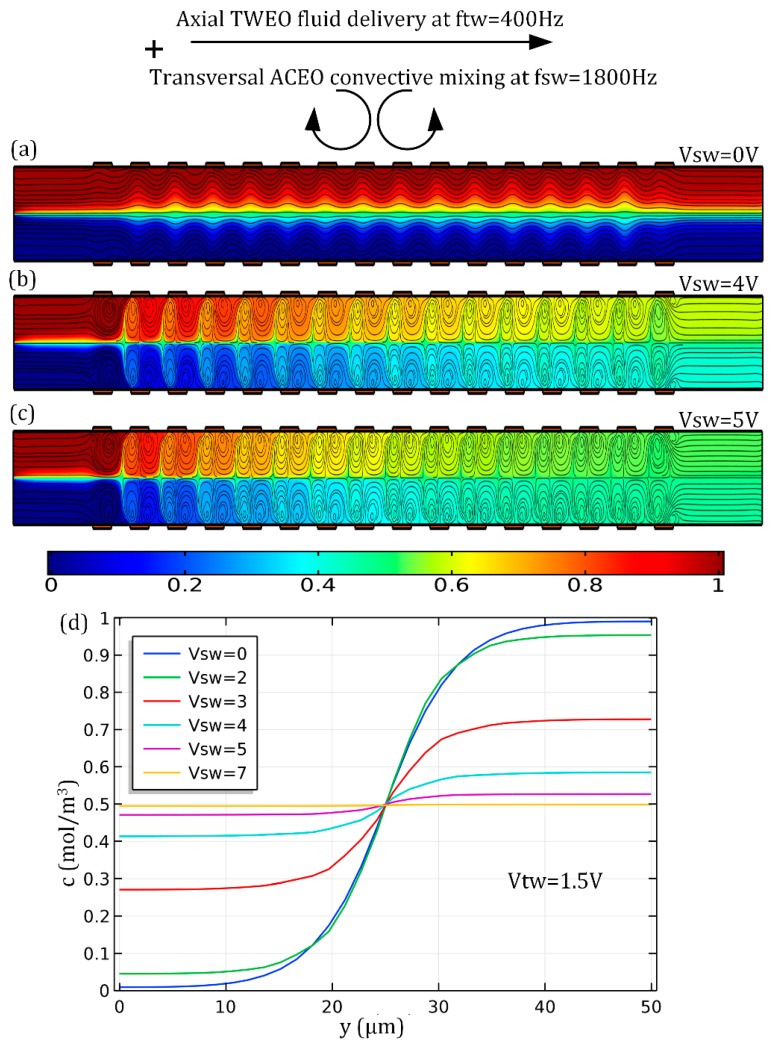
Simultaneous external-pump-free sample delivery and convective stirring completely driven by multifrequency induced-charge electroosmosis with a sufficient channel height of *Hc* = 500 μm compared to the lateral dimension *Wc* = 50 μm. (**a**–**c**) Sample treatment with MICEO under given *f_TW_* = 400 Hz, *f_SW_* = 1800 Hz and *V_TW_* = 1.5 V, a surface plot of analyte concentration and a streamline plot of MICEO flow field as a function of V_SW_ in the horizontal central plane of the fluidic channel; (a) *V_SW_* = 0 V where the interface between the two co-flowing laminar streams is mainly expanded by Brownian diffusion mechanism; (b) *V_SW_* = 4 V where both diffusion and lateral ACEO chaotic advection contribute to perturb the contact-interface; (c) *V_SW_* = 5 V where the strong EHD shear stress is main mechanism responsible for splitting and recombination of the central phase boundary. (**d**) Analyte concentration distribution along the channel centerline of the exit plane. In ((a–c), it is noteworthy that the length scale in the *y*-direction is arbitrarily extended twofold for enabling better visual clarification of MICEO-induced dual-functionality in simultaneous analyte pumping and mixing.

In the simulation analysis of the device dual-functionality, the actuating frequencies are still set as these ideal ones, that is, *f_SW_* = 1800 Hz and *f_TW_* = 400 Hz, and we fix the *TW* voltage amplitude at *V_TW_* = 1.5 V, so as to focus on the effect that the *SW* signal has on the pump and mixing performance. As shown in Figure 10a, in the absence of ACEO whirlpools with the supply of the *SW* voltage source turned off (*V_SW_* = 0 V), the electrolyte solution is indeed solely pumped by TWEO from the T-junction to the downstream outlet port, and the average transportation speed *u_x_* = 2.89 mm/s is totally determined by the *TW* signal. However, under this situation, the two-coflowing side-by-side laminar streams move parallelly along the channel length direction, and there is no apparent advective perturbation to the two-phase contact interface. Therefore, sample stirring, if there is any, is merely caused by molecular diffusion effect across the concentration gradient perpendicular to the phase boundary, giving rise to a humble mixing performance no more than 20% at the exit plane (Figure 10a).

On the contrary, on switching the *SW* signal on with voltage amplitude *V_SW_* = 4 V, a series of turbulent ACEO micro-vortices in opposite rotating directions takes place in the vicinity of the double-sided electrode array, being symmetric with respect to the horizontal centerline of the fluidic channel (Figure 10b). The lateral ACEO electrokinetic eddies distributed along the flow path intersect orthogonally with the laminar pump streamlines in the longitudinal direction induced by TWEO, resulting in the formation of double helical streamlines that move along the channel length direction until reaching the downstream outlet, where a liquid mixture of moderate mixing efficiency *γ* = 85.31% can be collected manually (Figure 10b). On increasing the *SW* voltage amplitude from 4 V to 5 V, although the TWEO pump flow rate keeps the same, the lateral electro-convective perturbation acting on the phase boundary becomes larger in strength, giving rise to a more helical flow profile and a better device mixing performance *γ* = 95.11% with an identical sample throughput (Figure 10c). 

As shown in Figure 10d, this can be reflected by the distribution of sample concentration gradient inside the outlet plane as well. To be specific, the lateral concentration profile varies from 0 mol/m^3^ to 1 mol/m^3^ quite sharply at *V_SW_* = 0 V (the blue line in Figure 10d), with the particle number density gradient similar to that at the channel inlet (not shown). As the *SW* voltage grows up gradually, the concentration profile has a propensity to become much flatter (from the green to the purple line in Figure 10d). What is more, as *V_SW_* further increases and approaches 7 V (the yellow line in Figure 10d), the concentration profile becomes almost a straight line oriented along the channel width direction and is of a constant value *c* = 0.5 mol/m^3^, that is, the surface-averaged analyte concentration in the inlet plane. 

In this sense, as the incoming liquid solution is continuously delivered by horizontal TWEO pump streamlines towards the channel exit, ACEO vortex flow field of sufficient actuating range exerts an effective hydrodynamic stress on the concentration gradient, which alternatively splits and recombines (SAR) the diffusing interfaces along the channel axial direction, that is, it serves as a category of field-induced chaotic advection mechanism similar to the previous passive SAR micromixer exploiting more complicated 3D channel structures [84]. Although the turbulent MICEO streaming induced by the hybrid AC signals promotes the molecule exchange rate across the diffusing phase interface by accelerating convective mass transfer along the channel width direction, the most prominent improvement of the proposed microdevice over those active micromixers reported in previous literatures is that there is no need for an external mechanical syringe to enable sample delivery in current fluidic mixer, since the liquid medium can be automatically pumped by TWEO originated by phase-shifted component of the applied voltage gradient.

**Figure 11:**
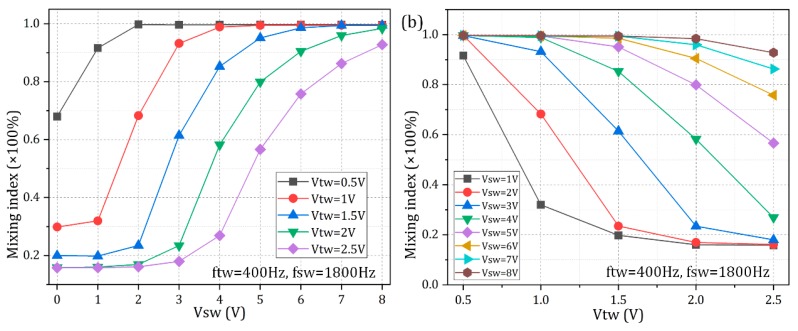
Quantitative characterization of the mixing efficiency of the dual-functionality MICEO microfluidic device, which makes use of pure nonlinear electrodynamic effect in double Fourier-mode AC fields. (**a**) *V_SW_*-dependence of the mixing index for different *TW* voltage amplitude; (**b**) *V_TW_*-dependence of the sample mixing performance under different *SW* voltage magnitude.

At last, it is essential to present some quantitative calculation data about the device dual-functionality and make a pertinent analysis. As shown in Figure 11a, within the framework of ideal working frequencies (*ftw* = 400 Hz, *fsw* = 1800 Hz), whatever the traveling wave signal is, the device mixing performance always enhances with the rise of the magnitude of the standing wave voltage, since the TWEO pump flow rate remains unchanged at a fixed *TW* signal while the lateral ACEO electroconvective perturbation reinforces by raising the *SW* voltage in this scenario. Besides, as displayed in Figure 11b, whatever the *SW* signal is, the mixing index at the outlet plane always decreases as voltage amplitude of the *TW* electric field increases. As for the second case, the vertical EHD shear stress on the interface keeps the same at a given *SW* voltage, while the horizontal pumping body force is made more potent by increasing the *TW* signal, and thereby, though the mixing index drops, the axial flow flux increases resulting in a higher sample throughput. To this end, it is quite convenient to regulate the ratio of mixing efficiency to the device throughput by adjusting the relative magnitude of the *SW* and *TW* voltage signal, implying a high degree of freedom control on the dual-functionality of the microfluidic chip in simultaneous analyte transport and convective mixing at the nanoliter scale.

### 3.7. Advantages and Limitations of the Method of MICEO

One distinguished trait of MICEO is that the electrokinetic flow profile is easily controllable by coordinating the voltage ratio between the *SW* and *TW* signal, as long as the ideal frequencies for ACEO and TWEO to operate separately have been confirmed by theoretical prediction. The convection mode can be made either more chaotic as ACEO predominates or more parallelly streaming when TWEO pumping of electrolytes is dominant. In addition, with appropriate voltage combinations, it is quite feasible to realize simultaneous delivery and convective mixing by exploiting a general device design into which a double-sided quarter-phase linear electrode array is embedded. What is more, induced-charge electroosmotic trapping performance of nanoparticles can be enhanced to great extent via utilizing the unique technique of MICEO introduced herein. 

Frankly speaking, in spite of these aforementioned advantages, there are still two limitations that potentially affect the actual performance of current method for practical on-chip applications: on one hand, since it has been reported that the physics of ICEO is mainly apt for driving fluid motion in the limit of dilute electrolyte, MICEO may malfunction in highly concentrated buffer solutions where ion overcrowding overwhelms ion overscreening inside the IDL on the ideally polarizable surface; on the other hand, since there is a 90° voltage phase shift between consecutively distributed metal strips in each repeating wavelength, it is inevitable to apply multilayer fabrication process if the configuration of a linear *TW* electrode track is to be utilized.

### 3.8. Guidelines on the Usage of MICEO for Improving Real Microfluidic Systems

According to the discussions in Section 3.7, if researchers want to make use of MICEO to improve their own microfluidic systems, they should at first make a judgement on whether their fluidic sample can survive in low-conductivity buffer solutions. As previously reported, ICEO is effective for liquid electric conductivity being no more than 0.02 S/m. Moreover, no ICEO fluid motion is observable on top of the electrode array in high-conductivity liquid buffers, when considering a shrinkage in IDL thickness and an enhancement in the steric effect with growing ionic strength. So, biological materials, it they require low conductivity buffers, can still be utilized in ICEO experiments. In this sense, ICEO is merely apt for handling low-conductivity solutions where the induced double layer can be fully developed. Even so, there are still many kinds of samples needing electrokinetic manipulation in dilute electrolytes, e.g., bacteria particles dispersed in tap water, whose content is of great importance for human’s physical health. Indeed, it is counterintuitive to deal with biological samples suspended in highly conductive fluids by electroosmotic streaming. By taking suitable pre-processing steps, however, this drawback can be alleviated to some extent. For example, resuspending the samples of bioparticles in low-conductivity sucrose media not only can maintain their viability, but solution conductivity is lowered to the level of DI water enabling an effective actuation of ICEO on polarizable surfaces as well. 

Once the issue of medium conductivity is addressed, it is then necessary to make use of the simulation model outlined in Section 2.2 and Section 2.3 to test the feasibility of the device design with distinct electrode patterns in practical on-chip sample handling. In the simulation study, researchers ought to take advantage of multifrequency phase-shifted AC sinewaves exerting on the discrete electrode array for inducing the phenomenon of MICEO, under the synergy of both in-phase and out-of-phase electrochemical polarizations and explore the effect that different voltage parameters would have on the resultant electroosmotic flow pattern. The optimized experimental parametric space at a given channel structure can be discovered as well for producing the anticipated helical streamlines with a prescribed axial-pump/transversal-mixing flow velocity ratio. In this sense, numerical prediction in advance with Equations (1)–(7) has the potential to help improve the success rate of realistic experiment in which MICEO is employed for a series of particle manipulation process. 

## 4. Conclusions

To summarize, we have presented results from both simulation analysis and experimental observation, to introduce a brand-new physical concept of multifrequency induced-charge electroosmotic (MICEO) slipping on ideally polarizable surfaces of a series of parallelly-placed metal strips. In MICEO, a phase-shifted hybrid AC voltage signal is imposed to a double-sided discrete electrode array arranged along channel sidewalls, and the time-averaged nonlinear Coulomb force within the induced-double layer, wherein the Debye screening charge has two components oscillating at distinct frequencies and spatial phase gradients, gives rise to simultaneous directed transport and convective mixing of the working fluid under a subtle combination of lateral ACEO turbulent perturbation and axial TWEO pumping motion. Our theoretical analysis and experimental validation demonstrate, for the first time, that induced-charge electroosmosis can be actuated by multi-frequency AC electric fields of different phase-transition characteristics at the same time. This kind of multiple Fourier-mode ICEO streaming is directly related to several exciting applications, including particle preconcentration, simultaneous sample delivery and stirring, as well as any subsequent biochemical analysis in microfluidics. The horizontal TWEO streamlines (rotating ACEO micro-vortices) are either profitable for pumping (mixing) or harmful for mixing (pumping), and our physical descriptions on multi-frequency ICEO above driving electrode arrays can guide the elaborate design of flexible electrokinetic frameworks to either strengthen or suppress them. The most salient feature about MICEO is its robust dual-functionality in unidirectional delivery and chaotic mixing of chemical analytes in a synergistic mode, and it is quite feasible to reconcile their relative importance by adjusting the ratio of standing and traveling wave voltage amplitudes. It is highly anticipated that multifrequency ICEO would actively stimulate the interdisciplinary research on nonlinear electrokinetics, analytical chemistry, and condensed matter physics in the broad context of microfluidics, nanofluidics, and lab-on-a-chip in the near future.

## Supplementary Materials

The following are available online at https://www.mdpi.com/2072-666X/10/7/447/s1, Video S1: Flow tracing experiment of MICEO.
